# Comparative Proteomics Reveals Cold Acclimation Machinery Through Enhanced Carbohydrate and Amino Acid Metabolism in Wucai (*Brassica Campestris* L.)

**DOI:** 10.3390/plants8110474

**Published:** 2019-11-06

**Authors:** Lingyun Yuan, Shilei Xie, Libing Nie, Yushan Zheng, Jie Wang, Ju Huang, Mengru Zhao, Shidong Zhu, Jinfeng Hou, Guohu Chen, Chenggang Wang

**Affiliations:** 1 Vegetable Genetics and Breeding Laboratory, College of Horticulture, Anhui Agricultural University, Hefei 230036, China; yuanlingyun@ahau.edu.cn (L.Y.); fyxsl@ahau.edu.cn (S.X.); 18720091@ahau.edu.cn (L.N.); Z18720097@ahau.edu.cn (Y.Z.); 17720092@ahau.edu.cn (J.W.); z19720182@ahau.edu.cn (J.H.); 17720095@ahau.edu.cn (M.Z.); sdzhuaau@ahau.edu.cn (S.Z.); houjinfeng@ahau.edu.cn (J.H.); cgh@ahau.edu.cn (G.C.); 2 Provincial Engineering Laboratory for Horticultural Crop Breeding of Anhui, Hefei 230036, China; 3 Wanjiang Vegetable Industrial Technology Institute, Maanshan 238200, China

**Keywords:** *Brassica campestris*, cold acclimation, proteomics, carbohydrate metabolism, amino acid

## Abstract

Limited information is available on the cold acclimation of non-heading Chinese cabbage (NHCC) under low temperatures. In this study, the isobaric tags for relative and absolute quantification (iTRAQ) were used to illustrate the molecular machinery of cold acclimation. Compared to the control (Cont), altogether, 89 differentially expressed proteins (DEPs) were identified in wucai leaves responding to low temperatures (LT). Among these proteins, 35 proteins were up-regulated ((and 54 were down-regulated). These differentially expressed proteins were categorized as having roles in carbohydrate metabolism, photosynthesis and energy metabolism, oxidative defense, amino acid metabolism, metabolic progress, cold regulation, methylation progress, and signal transduction. The fructose, glucose, and sucrose were dramatically increased in response to cold acclimation. It was firstly reported that aspartate, serine, glutamate, proline, and threonine were significantly accumulated under low temperatures. Results of quantitative real-time PCR analysis of nine DEPs displayed that the transcriptional expression patterns of six genes were consistent with their protein expression abundance. Our results demonstrated that wucai acclimated to low temperatures through regulating the expression of several crucial proteins. Additionally, carbohydrate and amino acid conversion played indispensable and vital roles in improving cold assimilation in wucai.

## 1. Introduction

Low temperature (LT) is a major determinant that limits the geographical distribution and growing season of plants [1]. Overwintering plants may increase their cold tolerance when exposed to low temperatures. This process is known as cold acclimation, which begins following exposure to low, non-freezing temperatures. Many studies have reported symptoms and metabolism changes in response to cold stress or chilling stress [2], including photoinhibition [3], carbohydrate accumulation [4], and signal transduction [5]. Wucai (*Brassica campestris* L. ssp. *Chinensis var*. *rosularis* Tsen et Lee), a member of the Brassicaceae family, is a semi-hardy non-heading Chinese cabbage (NHCC) that can safely overwinter in southern China and is an important winter leafy vegetable class [6,7]. However, there is little molecular information available regarding its cold acclimation. 

Cold acclimation is a complex biological process involved in the activation of physiological reactions and cold-related genes, such as the accumulation of osmotic substances, the regulation of lipid composition and hormone balance, etc. [8,9,10,11]. Research on cold acclimation has been reported for *Arabidopsis* [9], wheat [12], and barley [13]. In fact, due to the different genetic backgrounds, there are also significant differences in the cold acclimation ability of plants. The adaptation of plants to cold environments is regulated by strongly altered gene expression, which involves changes in the composition of the transcriptome, proteome, and metabolome [14]. In addition, some cold-responsive proteins undergo post-translational modifications, including phosphorylation, N-glycosylation, and ubiquitination, etc. [15]; The key aspects of protein function are determined by changes such as subcellular localization, stability, activity, or ability to interact with other proteins. The abundance of a protein is not only regulated by transcriptional but also by translational and post-translational levels. Consequently, the application of the proteome is crucial to our research, since proteins, unlike transcripts, are direct effectors of the plant stress response.

Proteomics, as a very powerful tool, is used to evaluate the functions of plant proteins under abiotic stress [16]. Therefore, isobaric tags for relative and absolute quantification (iTRAQ), a new quantification platform in proteomics that uses isotope labeling coupled with LC-MS/MS, was used in the present study owing to its high sensitivity and accuracy. In the present study, the differentially expressed proteins (DEPs) involved in cold acclimation and related physiological parameters, such as photosynthesis, carbohydrate content, amino acids types, and content, will also be determined to further elucidate the effects of altered protein abundance. 

## 2. Results

### 2.1. Primary Data Analysis and Protein Identification

**Figure 1:**
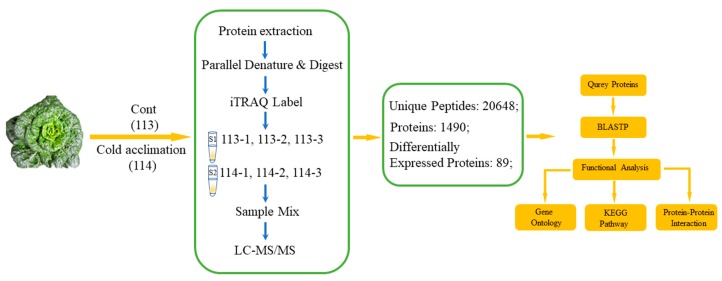
Schematic diagram for the identification of differentially expressed proteins via the isobaric tags for relative and absolute quantification (iTRAQ) method

**Figure 2:**
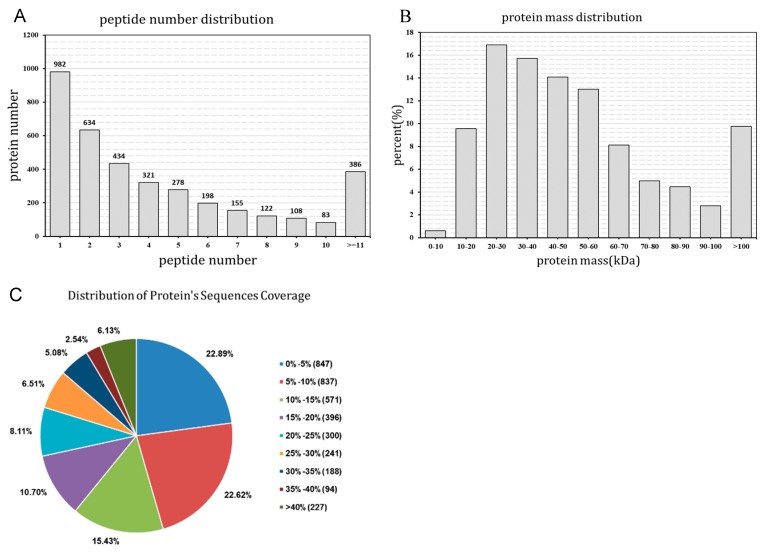
Identification and quantitative evaluation of identified proteins, including the distribution of the peptide numbers identified by Proteome Discoverer (**A**), the distribution of identified proteins according to molecular mass (**B**), and the distribution of protein sequence coverage (**C**).

The present study conducted a low-temperature-induced proteomic experiment by iTRAQ labeling in wucai leaves. After the samples were detected by LC-MS/MS, a total of 26,048 unique spectra were generated with 1490 proteins (score sequence HT > 0 and unique peptides ≥ 1) (Figure 1). The distribution of peptide number is shown (Figure 2A) with more than 73.5% of the proteins having at least two peptides. The protein mass distribution is shown in Figure 2B, which shows that approximately 99.4% of the protein mass was larger than 10 KDa, indicating a good coverage. The distribution of protein sequences covering more than 5% and 10% was 77.1% and 55.5%, respectively, thereby indicating high confidence (Figure 2C).

For this study, proteins with relative abundances of >1.2-fold were considered to be up-regulated proteins, whereas those with a relative abundance <0.83-fold were down-regulated at *p*-values of less than 0.05. A set of 89 differentially expressed proteins (DEPs) were identified under low temperature conditions compared with the control. Only significant proteins represented consistently in the biological replicates and these DEPs were used for further analysis.

### 2.2. Functional Cataloging of DEPs under Low Temperature

**Figure 3:**
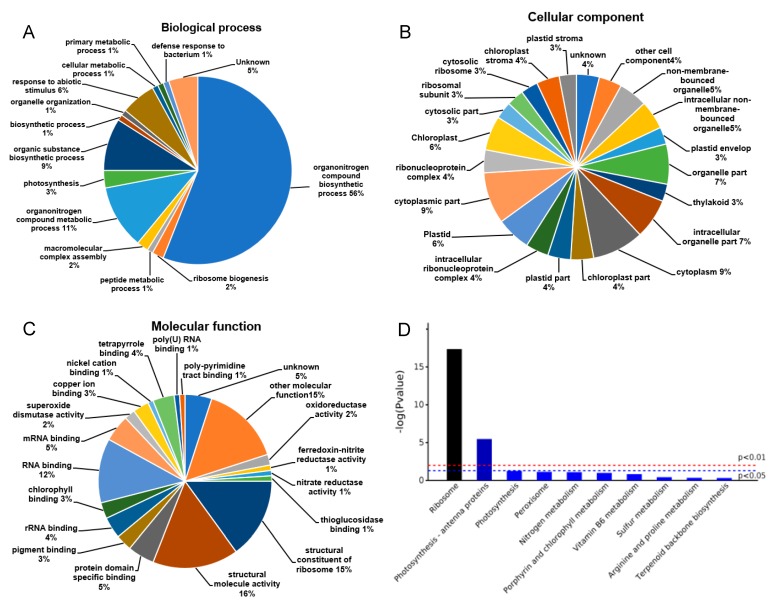
Classification of Gene Ontology (GO) in biological processes (**A**), cellular components (**B**), and molecular functions (**C**) and classification of pathway by the Kyoto Genomics and Genomics Encyclopedia (KEGG) in which the identified proteins involved are shown (**D**).

The functions of these DEPs were divided into several main groups based on their GO annotations, including biological processes (Figure 3A), cellular components (Figure 3B), and molecular functions (Figure 3C). As expected, some of the DEPs were identified in multiple groups. The analysis of biological processes suggested that the predominant pathways were the organonitrogen compound biosynthetic process and ribosome biogenesis, which accounted for 56% and 2%, respectively (Figure 3A). Most of DEPs were predicted to localize in the ribosome, plasmid, and chloroplast (Figure 3B). The most predominant molecular function was a structural constituent of the ribosome (15%), structural molecule activity (16%), and protein domain specific binding (5%). According to the Kyoto Genomics and Genomics Encyclopedia (KEGG) pathway analysis, most of the DEPs were enriched in the ribosome and photosynthesis-antenna proteins. The 89 DEPs identified were mainly involved in carbohydrate metabolism (6/89), photosynthesis and energy metabolism (11/89), oxidative defense (6/89), amino acid metabolism (4/89), metabolism (46/89), cold regulation (1/89), methylation (9/89), and signal transduction (6/89). Thirty-five out of the 89 DEPs showed increased (54 showed decreased) protein abundance-responding to cold acclimation when compared to the control. However, due to the types of samples, technical restraint, and limited reports, only 33 DEPs with clear protein names and functions were identified (Supplementary S1). The rest of the proteins were analyzed according to the mapped genes.

### 2.3. DEPs Involved in Different Metabolism Processes

#### 2.3.1. DEPs Involved in Carbohydrate Metabolism

**Figure 4:**
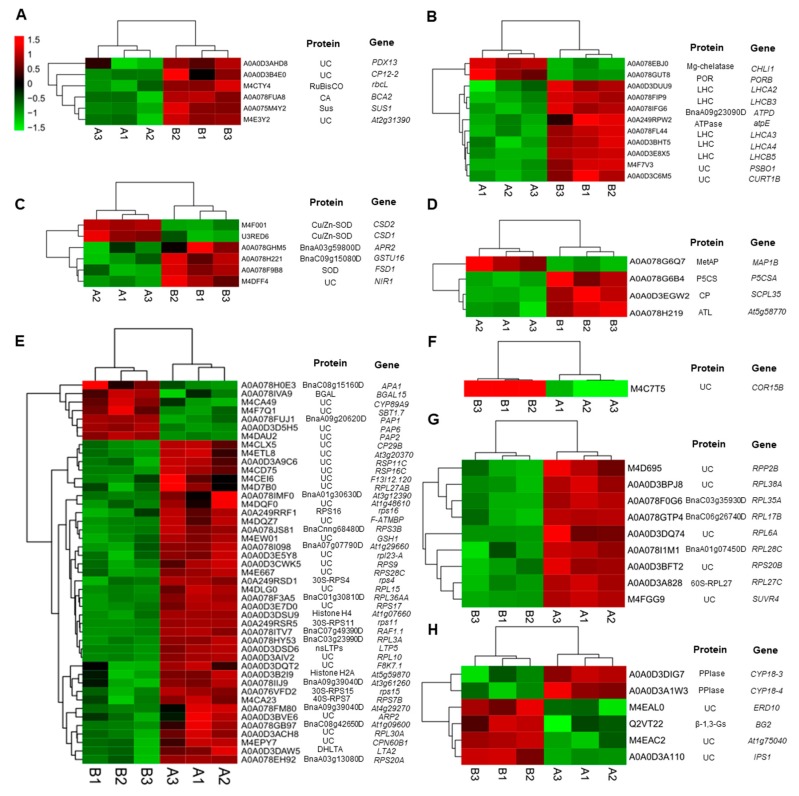
Heatmap of the 89 identified differentially expressed proteins (DEPs) at low temperatures (B1, B2, and B3) compared to the control (A1, A2, and A3). These proteins were classified into carbohydrate metabolism (**A**), photosynthesis and energy metabolism (**B**), oxidative defense (**C**), amino acid metabolism (**D**), metabolism (**E**), cold regulation (**F**), methylation (**G**), and signal transduction (**H**).

The present study identified some proteins with confirmed roles in carbohydrate metabolism. Compared to the control, proteins such as rubilose bisphosphate carboxylase large chain (RuBisCO, O03042), sucrose synthase (Sus, P49040), and carbonic anhydrase (CA, P42737) were up-regulated, and the abundance of three uncharacterized proteins mapped to the corresponding genes—*PDX13* (Q8L940), *At2g31390* (Q9SID0), and *CP12-2* (Q9LZP9)—also increased under low temperatures (Figure 4A).

#### 2.3.2. DEPs Involved in Photosynthesis and Energy Metabolism

Several critical proteins related to photosynthesis and energy metabolism were found to be up-regulated under low temperatures (Figure 4B). These DEPs included chlorophyll a/b binding protein (LHCs, Q9S7M0, Q9SY97, P27521, Q9SYW8, Q9XF89), ATP synthase epsilon chain (ATPase, P09468), and three uncharacterized proteins mapped to *PSBO1* (P23321), *ATPD* (Q9SSS9), and *CURT1B* (Q8LCA1), respectively. Low temperatures also caused decreases in the abundance of Mg-protoporphyrin IX chelatase (Mg-chelatase, P16127) and NADPH-protochlorophyllide oxidoreductase (POR, P21218), related to chlorophyll biosynthesis.

#### 2.3.3. DEPs Involved in Oxidative Defense

In the present study, some antioxidative proteins were involved in the cold acclimation of wucai. Three superoxide dismutases (SOD, P21276, O78310, and P24704) were identified; One was up-regulated and the other two were down-regulated in response to low temperatures (Figure 4C). One protein with putative glutathione S-transferase activity (Q9XIF8) was up-regulated. The abundance of the two proteins with oxidoreductase activity, mapped to *NIR1* (Q39161) and *APR2* (P92981), was also enhanced. 

#### 2.3.4. DEPs Involved in Amino Acid Metabolism

Amino acids, as precursors to, and constituents of proteins, play important roles during extensive metabolic shifts under low temperatures. Some enzymes in amino acid metabolism such as carboxypeptidase (CP, Q9LEY1), delta-1-pyrroline-5-carboxylate synthase (P5CS, Q9LEY1), and alkyl transferase (ATL, Q56Y11) were up-regulated under low temperatures, while methionine aminopeptidase (MetAP, Q9FV52) was down-regulated (Figure 4D).

#### 2.3.5. Metabolism Related Proteins 

The present study showed that low temperature induced significant changes in nucleic acid metabolism and ribosomal proteins (Figure 4E). Most DEPs were classified as being involved in metabolic processes with molecular functions such as translation or binding. Several of them were identified or mapped to 30S ribosomal proteins (P56805, P56806, P56799, and P56802), 40S ribosomal proteins (Q9M885), or 60S ribosomal proteins (P51419). The proteomic analysis showed that most of them were down-regulated in response to low temperatures. It was found that three transcription factors, which belonged to the ZIP family, were up-regulated. Although most of them were uncharacterized, they were mapped to *PAP1* (O81439), *PAP2* (O49629), and *PAP6* (Q9LW57) genes.

#### 2.3.6. Cold Regulated Protein

Our study showed a clear induction of cold-regulated proteins during cold acclimation in wucai. One uncharacterized protein mapped to *COR15B* (Q9SIN5) was found, which was up-regulated in response to low temperature (Figure 4F).

#### 2.3.7. Proteins Involved in Methylation

Methylation alteration represents an important mechanism through which plants are able to quickly adjust protein expression in response to cold acclimation. According to GO annotation, nine proteins are involved in the methylation progress, which were all down-regulated at low temperatures (Figure 4G). Most of them were mapped to 60S ribosomal proteins (P51419, Q9SF53, P51413, Q9M0E2, O22860, and Q9FZ76), whereas *SUVR4* (Q8W595), mapped to Histone-lysine N-methyltransferase, showed the same change direction.

#### 2.3.8. Proteins Involved in Signal Transduction

Several proteins are involved in the stimulation of cold acclimation. In the present study, the abundance of beta-1,3-glucanases (ß-1,3-Gs, P33157) increased while two peptidyl-prolyl cis-trans isomerases (PPIase, P34790, Q42406) were induced to down-regulate under low temperatures (Figure 4H).

### 2.4. Analysis of Carbohydrate Content, O_2_^−^ Content, H_2_O_2_ Content, and MDA Content

**Figure 5:**
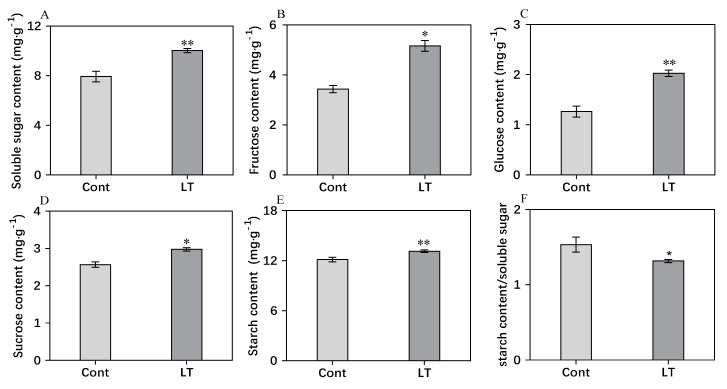
Carbohydrate contents and starch/soluble sugar ratio under low temperature and control conditions in wucai leaves. The amounts of Soluble sugar (**A**), Fructose (**B**), Glucose (**C**), Sucrose (**D**), Starch (**E**) and Starch content/soluble sugar (**F**) are shown. * represents significant at *p* < 0.05, ** represents significant at *p* < 0.01.

In order to estimate the carbohydrate content responding to cold acclimation, various sugar contents were determined. According to Figure 5, the contents of soluble sugar, glucose, sucrose, fructose, and starch showed significant increases under low temperatures. Compared to the control, soluble sugar (Figure 5A), fructose (Figure 5B), glucose (Figure 5C), and sucrose (Figure 5D) were significantly accumulated, which were increased by 20.9%, 33.4%, 37.6%, and 13.6%, respectively, at low temperatures. The starch content (Figure 5E) showed a slight increase of 7.7% under low temperatures. The starch/soluble sugar ratio exhibited a declining trend under low temperatures, decreasing by 13.7% when compared to the control. 

**Figure 6:**
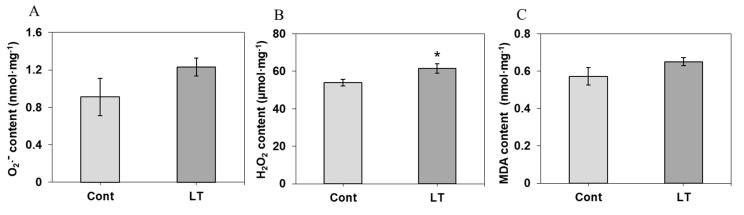
O_2_^−^ content, H_2_O_2_ content, and MDA content under low temperature and control conditions in wucai leaves. The contents of O_2_^−^ (**A**), H_2_O_2_ (**B**) and MDA (Malondialdehyde) (**C**) are shown. * represents significant at *p* < 0.05, ** represents significant at *p* < 0.01.

As shown in Figure 6A,B, after the cold accumulation, the contents of O_2_^−^ and H_2_O_2_ increased, and only the H_2_O_2_ content changed significantly, increasing by 13.9%. The MDA (Malondialdehyde) content was not changed significantly after cold acclimation.

### 2.5. Analysis of ATPase Activity and ATP Content

**Figure 7:**
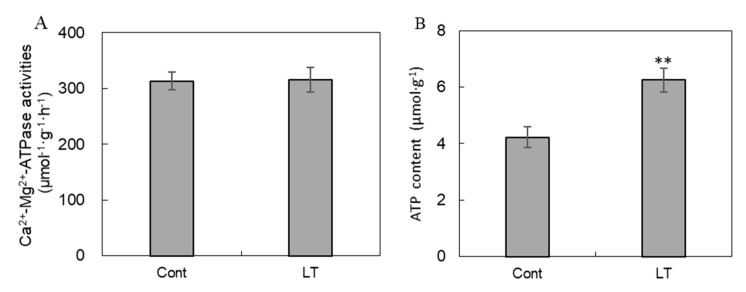
ATPase enzyme activities and ATP content under low temperature and control conditions in wucai leaves. The activity of Ca^2+^/Mg^2+^-ATPase (**A**) and ATP content (**B**) are shown. * represents significant at *p* < 0.05, ** represents significant at *p* < 0.01.

In our study, Ca^2+^/Mg^2+^-ATPase activities (Figure 7A) showed no differences under low temperatures, while ATP content was remarkably increased by 48.0% in response to low temperatures (Figure 7B).

### 2.6. Analysis of Chlorophyll (Chl) Content and Chlorophyll Fluorescence Parameters

According to Table 1, the total Chlorophyll content and Chl a/b ratio showed no distinct differences under cold stress conditions compared with the control. Interestingly, absorbed energy flux (ABS/RC) and dissipated energy flux (DI_o_/RC) were slightly higher under low temperatures compared with the control. They increased by 11.2% and 6.1%, respectively, whereas the electron transport flux (ET_o_/RC) decreased by 17.9%. The trapped energy flux (TR_o_/RC) did not show a significant difference under low temperature conditions. Significant increases occurred in the performance indexes including the response of the performance index was calculated on an absorption basis (PI_abs_) to cold acclimation. Compared with the control, low temperatures led to increases in PI_abs_ and PI_total_, which were increased by 7.1% and 11.3%, respectively.

### 2.7. Analysis of Free Amino Acids

Among the nonessential amino acids, aspartate, serine, glutamate, and proline contents were higher at low temperatures than under control conditions (Table 2). Glutamate and proline contents were increased to 3.71-fold and 4.27-fold under cold compared with control conditions. Under low temperatures, the contents of alanine and histidine showed no difference, while glycine, tyrosine, and arginine contents were reduced compared with the control. Among the essential amino acids, only the threonine content significantly accumulated, which increased by 1.92-fold compared with the control. Valine, methionine, leucine, phenylalanine, and lysine contents were remarkably decreased in response to cold acclimation, while cysteine and isoleucine contents showed no difference.

### 2.8. RT-PCR Analysis of Genes for Some DEPs

**Figure 8:**
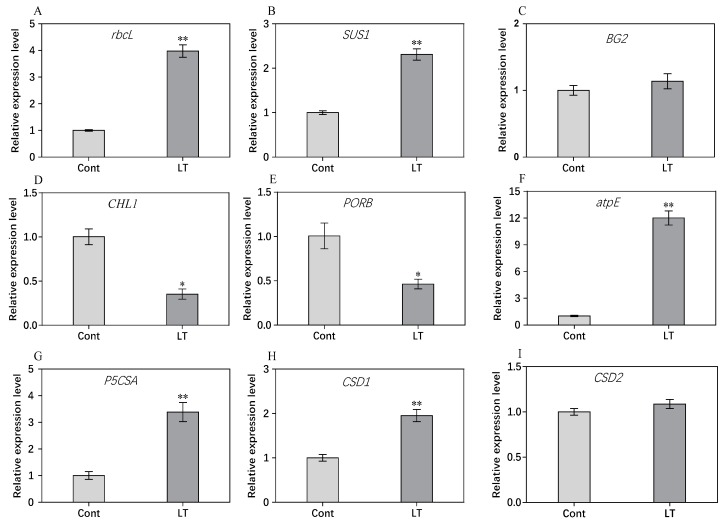
Transcriptional level analysis of nine genes encoding DEPs at low temperatures compared to control conditions. The transcriptional expression of the *rbcL* (**A**), *SUS1* (**B**), *BG2* (**C**), *CHLI1* (**D**), *PORB* (**E**), *atpE* (**F**), *P5CSA* (**G**), *CSD1* (**H**) and *CSD2* (**I**). * represents significant at *p* < 0.05, ** represents significant at *p* < 0.01.

To evaluate the correlation between mRNA and protein levels, nine of the DEPs were selected by RT-PCR. As shown in Figure 8, the results showed that the expression levels of *rbcL*, *SUS1*, *atpE*, *P5CSA,* and *CSD1* increased when exposed to low temperatures. Low temperatures resulted in decreases in the expression of *CHLI1* and *PORB*. The expression levels of *BG2* and *CSD2* exhibited no significant difference between low temperature and control conditions. A comparison of the expression patterns at the RNA and protein levels indicated that the transcriptional expression patterns of six genes were consistent with their protein expression abundance, whereas the other three genes displayed poor consistency between the transcriptional and translational levels in response to low temperature when compared to control conditions. It has been proven that the changes in the transcription level in gene expression often do not match the changes at the protein level.

## 3. Discussion

In cold acclimation, plants acquire tolerance based on prior exposure to low temperatures, however, various plant species differ in their ability to confront cold stress, which is governed by appropriate alterations in their metabolism, physiology, and growth [9]. Thus, understanding the involvement of these key proteins in cold acclimation can provide valuable insight for devising strategies to improve plant performance. Here, a comparative proteomics analysis of leaf soluble proteins successfully identified 89 DEPs. In the present study, we focused on the analysis of these DEPs, which are related to cold acclimation.

Our results suggest that the accumulation of osmoprotectants could be one strategy adopted for cold acclimation in wucai. The abundance of carbohydrate proteins, such as RuBisCO, CA, and Sus, was enhanced under low temperatures. In our study, we found that the up-regulation of RuBisCO was associated with increased photosynthesis (higher PI_abs_ and PI_total_) (Table 1). Increased abundance of RuBisCO was also a reflection of increased photorespiration resulting from reduced CO_2_ efflux, which was reflected by the upregulation of CA. CA is a zinc metalloenzyme that catalyzes the reversible hydration of CO_2_ and increases the inter-conversion between CO_2_ and bicarbonate (HCO_3_^-^) in living organisms [17]. It plays an important role in photosynthetic carbon assimilation [18]. Similarly, greater expression of Sus at the mRNA and protein levels was observed under cold conditions, which aimed at increasing the sucrose level as the main source of energy for various biochemical processes [19]. In the present study, the increased sucrose content not only facilitated revival as an osmolyte, it also played a nutritive role under natural conditions [20]. In the present study, the soluble sugar, fructose, sucrose, and glucose contents were distinctly increased under low temperatures, which indicated that in order to adapt to the low temperature environment, plants began to accumulate carbohydrates. Enhanced soluble sugars could resist cold damage to plant cells in a variety of ways, including serving as osmolytes or nutrient supplements or by interacting with the lipid bilayer [21]. Hu and Hou [22] reported that low temperatures led to a decrease in fructose content in non-heading Chinese cabbage, which was inconsistent with our result under low temperatures. The difference might be from different variants of wucai and baby bok choy in non-heading Chinese cabbage. This is the first study to report accumulated sugar types in wucai in response to cold acclimation. Moreover, the increased sugars contributed to nutrient accumulation and flavor improvement in wucai leaves.

Light-harvesting chlorophyll a/b-binding protein is one of the most abundant proteins of the chloroplast in plants [23]. It accounts for approximately half of the chlorophyll involved in photosynthesis. It is normally associated with chlorophyll and xanthophylls and serves as the antenna complex, which absorbs sunlight and transfers the excitation energy to the core complexes of PSII in order to drive photosynthetic electron transport [23]. We found that the abundance of five LHCs subjected to low temperatures was enhanced (Figure 4B). On the contrary, some previous reports have shown down-regulation of Chl a/b binding genes under different abiotic stress conditions in rice and barley [24,25]. In the present study, higher expression levels of Chl a/b binding proteins under low temperatures were considered to keep the PSII antenna complex intact, ensuring its functional involvement in the photosynthetic capacity [25,26]. Another photosynthesis-related, uncharacterized protein was also up-regulated, putative to PsbO, which was speculated to stabilize the thylakoid membrane in response to cold acclimation. PsbO is an extrinsic PSII protein located on the luminal side of the thylakoid membrane that organizes a peripheral structure surrounding the oxygen-evolving center of PSII [27]. The most important physiological role of PsbO is to stabilize the binding of the Mn4Ca cluster, which is essential for oxygen-evolving activity [28]. PsbO is sensitive to ROS accumulation, and it could be released from PSII, leading to inactivation of the oxygen-evolving center [29]. This higher abundance of PsbO also, in turn, verified stable redox homeostasis in cells by the up-regulation of oxidative defense and osmolyte accumulation. Chlorophyll a fluorescence is non-destructive and highly sensitive and can be used to evaluate important PSII properties in antenna complexes, including energy capture, electron transport, and excitation energy dissipation [30]. Among these parameters, PI_abs_ is a multiparameter expression of light and the performance index. PI_abs_ takes into consideration the three main functional steps of photosynthetic activity by a PSII reaction center complex, namely light energy absorption, excitation energy trap, and conversion of excitation energy to electron transport [31]. The higher PI_abs_ in the present study exhibited that the PSII structural integrity was almost well-maintained under low temperatures. The increased ABS/RC referred to the active PSII RCs (Reaction Centers), which might be due to the inactivation of some PSII RCs, as reported earlier [32]. Regrouping of the antenna from inactive PSII RCs to active PSII RCs can also lead to such observed increments in ABS/RC [33,34]. In spite of the high ABS/RC content, a very high rate of effective dissipation (as evidenced by high DI_o_/RC) of unstrapped excitations (TR_o_/RC values) occurred due to the low level of electron transport per PSII RC, i.e., ET_o_/RC. This down-regulation may prevent excessive reduction of the electron transport chain and promote the dissipation of excess energy to minimize photo-oxidative damage of the thylakoid membrane. Although chlorophyll biosynthesis related enzyme Mg-chelatase and POR expression abundance and their transcript levels were all down-regulated, the chlorophyll content and Chl a/b ratio were not significantly altered by low temperatures. This might imply that dynamic regulation existed in the pigment metabolism. We believe that low-temperature-induced photo-acclimation processes in wucai relied on alterations in the stoichiometry of photosynthesis proteins, and the relative amount/activity of the proteins was an important factor in photosynthesis. In our research, PSII stability, as recorded, could largely be attributed to higher expressions of putative PsbO and Chl a/b binding proteins to protect the thylakoid membrane. It was very useful in monitoring the dynamics of the photosynthetic capability in semi-hardy vegetables under low temperature. In addition, the abundance of ATP synthase and its transcript level were enhanced, accompanied by increased ATP content, whereas Ca^2+^/Mg^2+^-ATPase activity was not changed by low temperatures. This result indicates that Ca^2+^/Mg^2+^-ATPase played a vital role in improving the ATP content in wucai, thus maintaining cell metabolism. These results indicate that the up-regulation of photosynthesis and carbon metabolism were adaptive changes in order to meet the increased demand for carbon skeletons and energy. 

A previous study showed that amino acids could act as signaling molecules and permeates that regulate the transport of ions and promote detoxification and biosynthesis of proteins and nucleic acids [35]. Under LT, the biosynthesis of free and proteinogenic amino acids can be significantly affected as well. In the present study, the abundance of several proteins involved in the formation of amino acids, such as CP, P5CS and ATL, was enhanced (Figure 4D). The proline content at low temperature was increased 4.27-fold compared with the control, which was attributed to the higher glutamic acid content and P5CS abundance. It has been reported that the enhanced P5CS abundance with a higher transcript level could maintain the osmotic potential of the cell and hence save the plant from chilling [36]. Higher proline levels can maintain the structure and conformation of proteins by acting like molecular chaperones, protecting enzymes and other proteins from denaturation under extreme temperatures [37]. Under low temperatures, the methionine content decreased, which might be attributed to the decreased abundance of MetAP (Figure 4D). This result disagrees with previous reports, which reported an enhanced abundance of proteins involved in the formation of methionine in winter wheat [38]. The catabolism of threonine may be actively involved in the aspartase-derived amino acid pathway. In our study, threonine and aspartic acid contents were significantly accumulated (Table 2). Amino acid metabolism can increase the accumulation of compatible osmotic substances in plant stress resistance [39], which is also an important aspect of the nutritional quality of fruits and vegetables [40]. Our result firstly reported the main accumulated amino acid types during cold acclimation in wucai.

The increased abundance of antioxidative proteins plays an important role in cold acclimation and might maintain cellular redox homeostasis. This could be correlated with a more effective antioxidative enzymatic system for reactive oxygen species removal and, consequently, a lower susceptibility to oxidative stress (proved by the upregulation of superoxide dismutase), as has been shown previously under salt stress [41]. The total superoxide dismutase content was increased while the abundance of two Cu/Zn-SOD proteins declined. The up-regulated abundance of SOD and putative glutathione S-transferase facilitated the mitigation of the harmful effects of reactive oxygen species (ROS) and had a protective role in the cellular membrane system to resist oxidative damage due to low temperatures. They can also act as modulators and signal sensors for cellular signaling pathways to modify and maintain redox homeostasis [42]. 

Ribosome proteins (RPs) play important roles in protein synthesis and in maintaining the stability of ribosomal complexes, including small (RPS) and large subunits (RPLs). RPs not only have the function of stabilizing the ribosome complex and mediating the synthesis of the polypeptide but also have the in vitro function of ribose, such as responding to multiple stresses. For example, Under UV-B stress, RPL10 were regulated differently in a dosage- and time-dependent manner, RPL10A did not respond to UV-B when RPL10C was induced at a high UV-B intensity, and RPL10B was down-regulated [43]. Furthermore, the transcription levels of RPS15aA, RPS15aD, and RPS15aF were increased under temperature and mechanical stress. [44]. An enhanced accumulation of ribosomal proteins was observed in tolerant chickpea genotypes such as RPS2, RPS4, RPL33, and RPL24 [45]. However, in the present study, most of the ribosomal proteins, including 30S, 40S, and 60S, were down-regulated under low temperatures. Down-regulated proteins associated with protein biosynthesis and processing are the main consumers of ATP and nutrients, and this distribution allows translations to save energy and nutrients reasonably [39].

Changes in cell wall composition were associated with the up-regulation of proteins involved in cell wall modification-namely, beta-galactosidase (BGAL) was mapped to *BGAL15*. Up-regulation of one BGAL has been observed during the abscission of mature tomato orange fruits, suggesting that BGAL activity might play an important role during this abscission process [46]. Under low temperatures, up-regulated BGAL could lead to cell wall remodeling and expansion functioned as reported in *Arabidopsis* [47]. A putative cold-regulated protein, mapped to *COR15B*, was up-regulated in response to low temperatures, which showed a clear induction of cold-regulated proteins during cold acclimation. It was verified that *COR15B* could be induced by cold, abscisic acid and salt stress, indicating it could protect the membrane structure [48]. Some stress-related proteins were also responsive to low-temperature stress. The ß-1,3-Gs displayed enhanced protein abundance under low temperatures (Figure 4H), although its transcript level was not affected. It has antifreeze activity in the apoplastic space, which means that it is able to inhibit the recrystallisation of intercellular ice and even prevent the formation of intracellular ice [49]. Additionally, they are involved in signal transduction during cold stress [50]. According to proteomics data, by interacting with different processes (such as ROS sensing and defense), the abundance of the stress responsive gene *ERD10* was also increased to regulate ROS involved in temperature stress tolerance or acclimation in plants. [51].

Interestingly, epigenetic regulation was also found in the cold acclimation of wucai. Epigenetic modifications of DNA represent important mechanisms by which organisms can rapidly regulate gene expression in response to changes in environmental conditions, including heat stress [52]. Compared with the control, the methylation rate continues to decrease as the cold treatment time increases in cotton (*Gossypium hirsutum*). [53]. When it subsequently grows under normal conditions, it may recover but never return to its original level [54]. The level of methylation is dynamically altered under cold stress. Liu et al [55] found that in cold-acclimated *Brassica rapa*, some genes experienced increased methylation, while some were reduced. In our study, several proteins involved in the methylation progress were down-regulated, such as some ribosomal proteins and SUVR4. SUVR proteins, as histone methyltransferases, are suggested to be involved in the regulation of rRNA expression, thereby directing DNA methylation [56]. Thus, we suggest that DNA methylation plays an indispensable role in cold acclimation in wucai.

## 4. Conclusions

**Figure 9:**
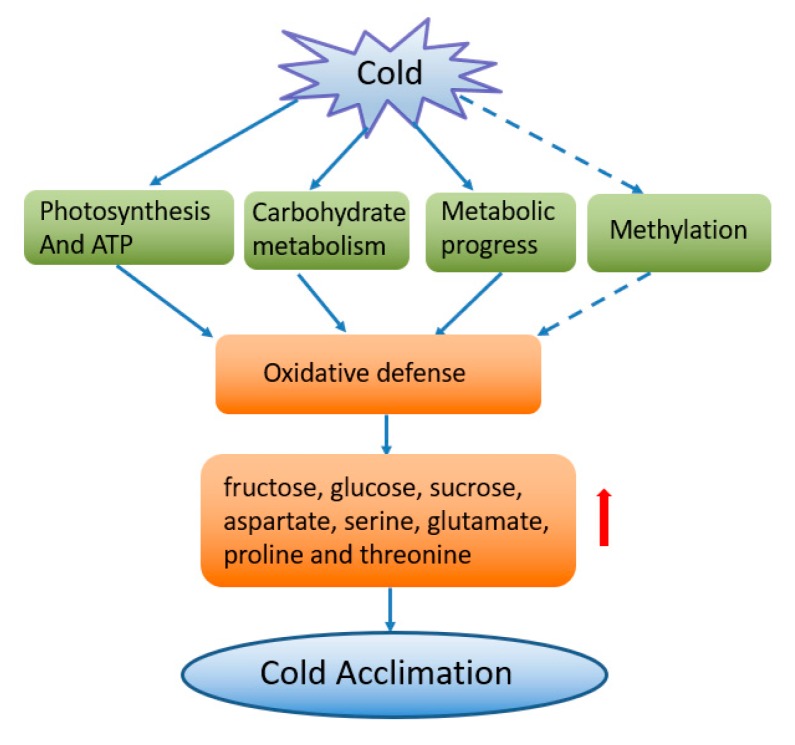
Schematic diagram of proteins involved in cold acclimation in wucai. Plants enhanced cold tolerance by the regulation of several crucial pathways, and led to the alteration of redox homeostasis. It induced soluble sugars and amino acid accumulation, thus facilitating cold acclimation. The solid arrows indicate known activation and the (….) indicate unclear effects. The red arrow indicates an increase in the osmolyte content.

Our results emphasize the roles of proteins involved in various pathways such as carbohydrate metabolism, photosynthesis and energy metabolism, oxidative defense, metabolic progress, and methylation (Figure 9). These data showed that cold acclimation in wucai is potentially characterized by the up-regulation abundance of some crucial proteins, such as RuBisCO, Sus, CA, ATPase, LHC, SOD, P5CS, putative COR, and PsbO. Down-regulated ribosomal proteins and methylation related proteins were also involved in cold acclimation. Physiological parameters exhibited that carbohydrate contents, including fructose, glucose, and sucrose were dramatically accumulated during cold acclimation. Aspartate, serine, glutamate, proline and threonine were the main accumulated amino acids, which played vital roles in stress acclimation and provided flavor and nutrient quality in wucai. Our research could be helpful for future research and further characterization of stress responses aimed at the cold acclimation of vegetable varieties.

## 5. Materials and Methods

### 5.1. Growth Condition 

W12-7 was a conventional germplasm and genetically stable germplasm that has been selected for multiple generations. It was selected as the experiment material. The seeds were provided by the Vegetable Genetics and Breeding Laboratory of Anhui Agricultural University. Seeds were sown in soil-less substrate (peat/vermiculite (volume) = 2/1) and then moved into a growth chamber, and the seedlings were maintained at 25 °C (day) and 18 °C (night) with a relative humidity of 70% and photosynthetic activity of 300 μmol·m^−2^·s^−1^ in a 14/10 h light/dark photoperiod. The seedlings were subjected to two treatments: control (Cont) treatment (25 °C /18 °C (day/night)) and low temperature (LT) treatment (10 °C /3 °C (day/night) for 10 days). They were arranged in a completely randomized design which was repeated in triplicate. Each repetition was treated as a block for a total of 3 blocks. The blade of the third fully expanded leaf from the center of each plant was sampled. The samples were immediately frozen in liquid nitrogen and frozen at −80 °C for physiological measurements and protein and RNA extraction.

### 5.2. Protein Preparation and iTRAQ Labeling

The schematic diagram was showed for the identification of DEPs via the iTRAQ method (Figure 1). Proteins in plant leaves were obtained by the trichloroacetic acid (TCA)/acetone method [56]. Every treatment was carried out with three biological replications. Protein digestion was based on the method of Wiśniewski et al. [57] and was labeled with 8-plex iTRAQ reagent. A unit iTRAQ reagent was added to 24 μL of isopropanol. At room temperature, the isobaric tag peptide was labeled for 2 h. Then, 200 μL of water was added to stop the reaction. The samples were labeled as 113-1, 113-2, 113-3, 114-1, 114-2, and 114-3. They were multiplexed and vacuum dried. Systematic steps were carried out as shown in Figure 1.

### 5.3. iTRAQ Analysis and Bioinformatic Analysis of Proteins

The iTRAQ-labeled peptide mixture was added to 4 mL of buffer A (25 mM KH_2_PO_4_ in 25% acetonitrile (ACN), pH 2.7)) by using strong cation exchange chromatography on an Agilent 1100 HPLC Purifier system (Agilent Technologies Inc., California, USA), which was then added to a 2.1 × 150 mm Agilent Zorbax Extend-C18 column. The peptide was eluted at 300 μL min^−1^ for 7 min with 5% buffer B (25 mM KH_2_PO_4_ and 1 M KCl in 25% ACN, pH 2.7) and then eluted in 5–60% buffer B for 20 min, 60–100% Buffer B for 2 min, and finally, 100% Buffer B for 1 min. The elution was carried out at 214 nm at each minute while collecting the fractions. The collected fractions were desalted by using a C-18 column and dried under a vacuum.

Each fraction was resuspended in buffer C (2% CAN, 0.1% formic acid (FA)). The precipitate was discarded, and 5 μL of the supernatant was applied to a C18-reversed phase analytical column (75 μm × 15 cm, 3 μm, 120 Å, ChromXP Eksigent). The sample was added to 5% buffer D at a linear concentration at 300 nL min^−1^ for 5 min (95% CAN, 0.1% FA), and 3–35% and 35–60% buffer D were added for 35 min, respectively. Then, 60–80% of buffer D was kept for 2 min, 5% of buffer D was added for 1 min, and finally, the solution was held in 5% of buffer D for 10 min. The LC-MS/MS analysis used a Q-Exactive mass spectrometer (Thermo Fisher Scientific, Waltham, MA, USA) coupled to Easy nLC (Thermo Fisher Scientific, Waltham, MA, USA) based on the method of Zhang et al. [58].

We searched the MS/MS spectra by using the Proteome Discoverer TM 2.2 (Thermo Scientific, Waltham, MA, USA) running on the Uniprot database. The search results used the following constraints: significance threshold: *p* < 0.05 (95% confidence); ion score or expected cutoff value < 0.05 (confidence level: 95%). The protein was quantified using the protein ratio as the median of the protein peptide. Median protein ratio normalization was used to calculate the peptide ratio.

An independent-sample t-test was used to determine a significant (*p* < 0.05) difference in the difference between the two treatments to analyze significant up- or down-regulation of DEPs. The threshold for the change rate of the low temperature compared to the control was >1.2 times or <0.83 times to define DEPs.

According to Yang (2013) [59], a bioinformatics analysis of proteins was performed. The annotations for these identified proteins were obtained by searching using the Uniprot database (January 28, 2018, 172 630 sequences). DEPs were classified according to biological functions using Gene Ontology (GO) terminology and mapped to the Kyoto Genomics and Genomics Encyclopedia (KEGG), which was used to identify active biological pathways.

### 5.4. Analysis of Carbohydrate Content, H_2_O_2_ Content, O_2_^−^ Produce Rate, and MDA Content

Freeze-dried leaf blades were sampled to estimate the carbohydrate content. The soluble sugar content and starch content were determined by the modified phenol-sulfuric acid method [60]. The content of glucose was determined using a biochemical kit (Cat#BC2500, Beijing Solarbio Science & Technology Co., Ltd, Beijing, China). 

The H_2_O_2_ content and O_2_^−^ content were measured by using the Solarbio reagent kit (Cat#BC3590 and Cat#BC1290, Beijing Solarbio Science & Technology Co., Ltd, Beijing, China). The content of MDA was measured in accordance with Hu et al. (2018) [61].

### 5.5. Analysis of ATPase Activity and ATP Content

Samples used to estimate the ATPase activity and ATP content were analyzed according to methods of Yao et al [62] and Stewart et al [63], respectively. The Ca^2+^/Mg^2+^-ATPase and ATP contents were measured using a biochemical reagent Kit (Cat#BC0960, Cat#BC0300, respectively; Beijing Solarbio Science & Technology Co., Ltd, Beijing, China).

### 5.6. Analysis of Chlorophyll Fluorescence Parameters 

A fast chlorophyll *α* fluorescence induction curve of the third fully expanded leaf from the center was made with a Pocket PEA (Plant Efficiency Analyzer, Hansatech, UK). Before measurement, leaves were subjected to dark conditions for 0.5 hours. The measured parameters were imported into PEA Plus 1.04 software for processing. The fast chlorophyll *a* fluorescence induction test parameter (OJIP-test) was generated using Biolyzer 3.0 software (Bioenergetics Lab., Geneva, Switzerland).

Various parameters related to PSII performance were calculated by the induction curve [64]. In this study, We calculated the following four parameters, which are involved in specific energy fluxes for single PSII reaction centers (RCs): electron transport flux (ET_o_/RC) = M_o_(1/V_J_) Ψ_o_, dissipated energy flux (DI_o_/RC) = (ABS/RC) − (TR_o_/RC).absorbed energy flux (ABS/RC) = M_o_(1/V_J_) (1/ψp_o_), and trapped energy flux (TR_o_/RC) = M_o_(1/V_J_). The performance index was calculated on an absorption basis (PI_abs_) as (RC/ABS) (ψp_o_/(1 − ψp_o_)) × (ψ_o_/(1 − ψ_o_)).

### 5.7. Determination of Free Amino Acids in Leaves

The determination of various free amino acid contents was slightly modified according to Aurisano et al. [65]. A total of 0.1 g of the freeze-dried sample was ground and then added to 2% sulfosalicylic acid. The pH was adjusted to 2.0 with 0.02 M HCl, and the mixture was centrifuged at 10,000 × *g* for 15 min at 4 °C. The content of each free amino acid was analyzed using an amino acid analyzer (Hitachi L-8900, Tokyo, Japan).

### 5.8. Analysis of the Expression Levels of Differentially Expressed Protein Related Genes

Based on the functional category and differential expression fold, nine genes were chosen by RT-PCR. Specific primers for differentially expressed protein-related genes were designed by Primer software version 5.0 (Premier Biosoft International, Palo Alto, CA, USA) (Table 3). Total RNA from two treatments was extracted using the kit (Takara Biomedical Technology Co., Beijing, China). AceQ qPCR SYBR GREEN Master Mix (Vazyme Biotechnology Co., Ltd., Nanjing, China) was used for RT-PCR analysis. The results were estimated using the 2-△△CT method. Every sample had three biological replicates. Three reactions were performed for all reactions of each sample, and the β-actin gene was used as an internal standard.

### 5.9. Statistical Treatment

The data performance was assessed using the mean ± SD of three replicates. A statistical significance (*p* < 0.05) analysis was performed using SAS software (SAS Institute, Cary, NC, USA) based on the use of Duncan’s multi-range test.

## Figures and Tables

**Table 1:** Chlorophyll (Chl) content and Chlorophyll Fluorescence index under control (Cont) and low temperature (LT) conditions.

DAP	Chl Content (mg∙g^−1^ FW)	Chl a/b Ratio	PI_abs_	PI_total_
Cont	2.84 ± 0.05 a	1.693 ± 0.08 a	14.68 ± 0.07 b	18.6 ± 0.21 b
LT	2.92 ± 0.53 a	1.701 ± 0.15 a	15.72 ± 0.22 a	20.70 ± 0.13 a
	ABS/RC	TR_o_/RC	DI_o_/RC	ET_o_/RC
Cont	0.9484 ± 0.0157 b	0.7648 ± 0.0043 a	0.1607 ± 0.0011 b	0.6273 ± 0.0140 a
LT	1.0542 ± 0.0181 a	0.7886 ± 0.0253 a	0.1705 ± 0.0036 a	0.5150 ± 0.0055 b

a, b letters represent significant at the *p* < 0.05 level under different treatments.

**Table 2:** Amino acid types and contents at Cont and LT.

Amino Acid (μg∙g^−1^)	Cont	LT
Nonessential amino acids		
Aspartate	**301.77 ± 5.32 b**	376.12 ± 12.65 a
Serine	113.52 ± 1.74 b	167.00 ± 5.24 a
Glutamate	24.33 ± 0.66 b	90.35 ± 3.78 a
Glycine	26.70 ± 0.62 a	24.64 ± 1.36 b
Alanine	180.84 ± 3.31 a	183.79 ± 7.14 a
Tyrosine	175.18 ± 3.28 a	119.03 ± 9.92 b
Histidine	26.87 ± 0.45 a	26.13 ±1.35 a
Arginine	222.83 ± 3.64 a	121.06 ± 2.75 b
Proline	16.94 ± 1.25 b	72.32 ± 4.88 a
Essential amino acids		
Cysteine	9.06 ± 0.85 a	6.58 ± 3.32 a
Valine	123.81 ± 2.80 a	91.33 ± 13.01 b
Methionine	42.01 ± 1.41 a	19.18 ± 9.02 b
Threonine	228.50 ± 3.48 b	439.02 ± 5.08 a
Isoleucine	99.21 ± 1.67 a	76.31 ± 13.84 a
Leucine	184.95 ± 3.13 a	145.08 ± 9.68 b
Phenylalanine	193.94 ± 3.11 a	149.24 ±10.16 b
Lysine	135.25 ± 2.21 a	123.84 ± 4.47 b

a, b letters represent significant at the *p* < 0.05 level under different treatments.

**Table 3:** Specific primers for differentially expressed protein related-genes.

Association No.	Gene Name	Forward Primer (5′→3′)	Reverse Primer (5′→3′)
XM_009127097.2	*ß-actin*	TGGGTTTGCTGGTGACGAT	TGCCTAGGACGACCAACAATACT
AY167977.1	*rbcl*	TATGCCTGCTTTGACCGAGA	GCAAGATCACGTCCCTCA
XM_009113306.2	*SUS1*	TTGGTGGAGAGTGGAGAGAAG	CTGGTACTGAAGCTCTGCCT
XM_009118181.2	*BG2*	GACGACCCATACTCTTACACA	TTCCAACGACCCTCCGCCTGAT
XM_009133530.2	*CHLI1*	CCACAGAAATCAACTCCAC	CATTATCATCACACCACCG
XM_009139412.2	*PORB*	TACACGGTGATGCATTTGGAC	GTCATCAAGCAACAACCTCGA
XM_018654732.1	*atpE*	AATGCTCTGGTGGTTAAGGGT	TCAACAGGTTCCCCAAGTACA
XM_009143589.2	*P5CSA*	AAGCAAGGTCGTTCAAG	TATTCCCACCTCAGCACCAA
XM_009149890.2	*CSD1*	TGCTGGCGATCTAGGAAACA	AGCCCTGAAGACCAATAATGC
XM_009142600.2	*CSD2*	CCGACAAAAGTGAGTGTTCGT	GGCAATAATGTTTCCCAGGTC

## Supplementary Materials

The following are available online at https://www.mdpi.com/2223-7747/8/11/474/s1, Supplementary S1: Classification of differentially expressed proteins in comparison to the control.
